# Research Progress on the Synthesis, Modification, and Applications of Microbial Biopolymers

**DOI:** 10.3390/polym17152053

**Published:** 2025-07-28

**Authors:** Shashi Kant Bhatia

**Affiliations:** 1 Department of Biological Engineering, College of Engineering, Konkuk University, Seoul 05029, Republic of Korea; shashikonkukuni@konkuk.ac.kr; 2 Institute for Ubiquitous Information Technology and Applications, Seoul 05029, Republic of Korea

Microbe-based polymers are considered a suitable alternative to conventional petroleum-based polymers for various industrial applications such as packaging, drug delivery, and tissue engineering [1,2]. Various biopolymers, such as polyhydroxyalkanoates (PHAs) and exopolysaccharides (EPSs), are produced by microbes as part of their metabolic processes [3,4,5]. These biopolymers act as a reserve food material for microbes and protect them from harsh environments, and are also explored for their diverse environmental, medical, and industrial applications due to their biodegradability, biocompatibility, and tuneable properties [6]. The growing global emphasis on reducing plastic waste and achieving the Sustainable Development Goal (SDG) of carbon neutrality has forced research into microbial biopolymer production as part of the broader effort towards a circular economy. Recent advancements in microbial polymer production are covered in this editorial (Figure 1).

Selection of biopolymer producers: Microbes have the capability to utilise various organic wastes (lignocellulosic biomass, sludge waste, whey, waste oil, etc.) as a carbon and nitrogen source for the growth and production of biopolymers [7]. *Ralstonia eutropha* is well known to accumulate polyhydroxybutyrate (PHB), while *Pseudomonas* sp. produces medium-chain-length PHAs (*mcl*-PHAs), and *Halmonas* sp. can grow under halophilic conditions and produce PHB without the need for any sterilisation [8,9]. Microbes such as *Halomonas* and *Pseudomonas* also coproduce EPSs under different nutrient-imbalanced conditions [10]. Recently, Sanchez et al. isolated a *Bacillus thuringiensis* strain able to accumulate PHB at a C/N ratio of 30 [11]. There is a need to identify and examine new microbes able to consume a wide range of carbon sources and produce higher amounts of polymers that possess unique properties. The feast and famine approach is frequently used for the selection of microbial cultures able to accumulate a high content of PHA [12]. Consortia engineering has also been explored by researchers to extend the substrate utilisation and provide precursors for polymer synthesis. In one study, Zhu et al. engineered artificial consortia of *Escherichia coli* MG1655 and *Pseudomonas putida* KT2440, finding that engineered *E. coli* utilise xylose and produce acetic acid and fatty acids, which are further used by *P. putida* and produce *mcl*-PHAs [13]. Other main breakthroughs in microbial biopolymer production have been the use of genetic engineering to enhance yield, produce copolymers, and modulate cell size for the high accumulation of PHAs [14,15]. Poly(3-hydroxybutyrate-*co*-3-hydroxyhexanoate) [P(3HB-*co*-3HHx)] is a PHA copolymer that improves the mechanical properties of P(3HB), but genetic modifications often reduce productivity. To overcome this, native oil utilising *Cupriavidus* strains was screened by Lim et al., and strain Oh_1 was identified as a high PHA producer. The engineered Oh_1/phaCRaJPa strain showed the highest 3HHx content (27.2 mol%) and PHA yield (48.93 g/L), with improved molecular weight compared to genetically disrupted strains. This enhancement was linked to the strong expression of *phaCRa* and *phaJPa* genes and upregulated *β*-oxidation pathways, offering a promising approach for high-quality P(3HB-*co*-3HHx) production without major genetic modifications [16]. Moreover, engineered strains may perform well under controlled laboratory conditions, but scaleup in industrial settings can present unforeseen challenges, such as contamination and competition from non-engineered strains. These limitations emphasise the need for more robust strategies to combine genetic engineering with process control for reliable, large-scale production.

Feedstock utilisation and economic viability: A central challenge in microbial biopolymer production is the high cost of raw materials (40% of the total cost), which limits the economic feasibility of large-scale production [17]. The use of low-cost and renewable feedstocks, such as lignocellulosic biomass and industrial waste, has been a focus of recent research for polymer production. However, the pretreatment of lignocellulosic materials, which is necessary to release fermentable sugars, remains a costly and energy-intensive process. The use of other waste such as waste cooking oil (WCO) is gaining interest as it can support *mcl*-PHA production, but its low solubility in media is the main issue [18]. Similarly, the valorisation of industrial wastes, such as paper and pulp industry by-products and waste-activated sludge, has emerged as a viable strategy to lower production costs. The use of methanotrophs for PHA production using methane is also receiving attention, but low productivity is the main barrier [19]. A two-step process (anaerobic and aerobic) is also used in which feedstock is first converted into volatile fatty acids anaerobically and then converted into PHAs aerobically [20]. Nevertheless, the challenge remains to ensure a consistent and reliable supply of such waste streams, as fluctuations in waste availability and composition can lead to variability in biopolymer production.

Technological advancements in cultivation: Various methods of cultivation, such as batch, semicontinuous, continuous, membrane bioreactors, and two-stage fermentation, have been reported based on feedstock and strains used. Batch fermentation is a laborious and time-consuming process, while the continuous process is easy and ensures continuous PHA production. Traditionally, pure cultures are preferred for PHAs due to their controlled growth conditions; recent studies have indicated that mixed microbial cultures (MMCs) are more effective for biopolymer production from waste streams [21]. MMCs from wastewater treatment plants can not only produce significant amounts of PHAs, but also adapt better to variable feedstocks than pure cultures. This adaptability is particularly important when using unconventional feedstocks, such as industrial or municipal waste, which may fluctuate in composition. However, the major challenge with MMCs is controlling microbial population dynamics to ensure stable and high-yield production over time. Studies have suggested that MMC performance can be unpredictable, as different microbial species may dominate under varying environmental conditions, leading to fluctuations in polymer yield and quality. Therefore, optimising the conditions under which MMCs operate, such as reactor design and feedstock composition, remains critical for the success of this approach. One key limitation of current reactor designs is their sensitivity to fluctuations in feedstock quality and microbial growth conditions. Studies have shown that small variations in pH, temperature, or nutrient availability can lead to significant drops in biopolymer production. Thus, future technological advancements should focus on incorporating real-time monitoring and control systems within bioreactors to maintain optimal conditions. These innovations are critical for scaling up biopolymer production and ensuring consistent product quality.

Modification and fabrication of biopolymers: Microbial biopolymers, with their inherent functional groups, are highly amenable to chemical modification, enhancing their material properties for specific applications. Wu et al. functionalised PHA nano beads with antimicrobial peptides, and the produced material was effective in inactivating pathogens [22]. Blending PHAs with other polylactic acid (PLA) and TiO_2_ has been shown to improve crystallinity, thermal stability, and memory properties [23]. However, these modifications can sometimes negatively impact the biodegradability of the biopolymers, as blending with non-microbial polymers may result in slower degradation rates under environmental conditions. Thus, careful consideration must be given to the trade-offs between enhanced material properties and environmental sustainability. In biomedical applications, polysaccharides can also be blended with other natural and inorganic materials to improve their properties and applications. However, one critical challenge in these applications is ensuring that chemical modifications do not affect the biopolymer’s interaction with biological tissues, which could compromise their performance in medical devices.

Biodegradation of biopolymers: Microbial biopolymers have found extensive applications in various sectors, including packaging, healthcare, and environmental sustainability. PHAs, due to their biodegradability, have been widely promoted as alternatives to traditional plastics. Research has shown that biobased polymers can be fully degraded by microbial action in marine environments, making them ideal for addressing ocean plastic pollution. However, the rate of degradation depends heavily on the microbes involved, the type of polymer, and environmental factors such as temperature and microbial activity. Volvo et al. studied the degradation of different polymers in field soil, and the degradation rate was ranked in the order P(3HB/4HB)  >  P(3HB/3HHx)  >  P(3HB/3HV)  >  P(3HB); different microbes were involved in the degradation of various polymers [24]. The degradation of P(3HB) was uniquely carried out by bacteria from the genera *Mitsuaria*, *Chitinophaga*, and *Acidovorax*, which did not degrade the other three PHA types. *Roseateles depolymerans*, *Streptomyces gardneri*, and *Cupriavidus* sp. were specific to P(3HB/4HB) degradation. *Roseomonas massiliae* and *Delftia acidovorans* selectively degraded P(3HB/3HV), while *Pseudoxanthomonas* sp., *Pseudomonas fluorescens*, *Ensifer adhaerens*, and *Bacillus pumilus* were specific to P(3HB/3HHx) degradation [24]. In healthcare, microbial biopolymers are used in wound healing, drug delivery, and tissue engineering due to their excellent biocompatibility. However, challenges remain in controlling the rate of degradation in biological environments, as overly rapid degradation can reduce their efficacy in long-term therapeutic applications.

Techno-economic analysis: While microbial biopolymers have shown great promise, their commercial viability is still hampered by high production costs. The PHA production cost varies depending on the feedstock used (glucose (USD 8.6/kg), sucrose (USD 3.7–11.9/kg), dairy whey (USD 5.1–7.9/kg), waste glycerol from biodiesel production (USD 2.0–2.6/kg)) and remains significantly higher than that of petroleum-based plastics (USD 0.9–1.0/kg) [25,26]. One potential solution to lower the production cost is the integration of waste valorisation strategies and co-producing other products (EPS, biohydrogen, etc.), which can lower overall costs and contribute to a circular economy. However, the inconsistent availability of waste streams remains a key limitation to this approach.

Microbial biopolymers represent a promising, sustainable alternative to conventional plastics, with applications across various industries. Recent advancements in genetic engineering, process optimisation, and waste valorisation have significantly improved the feasibility of microbial biopolymer production. However, challenges related to production cost, scalability, and material performance must be addressed to make these biopolymers commercially viable. Future research should focus on optimising microbial cultures, enhancing biopolymer modifications, and integrating sustainable feedstock strategies to unlock the full potential of microbial biopolymers.

## Figures and Tables

**Figure 1:**
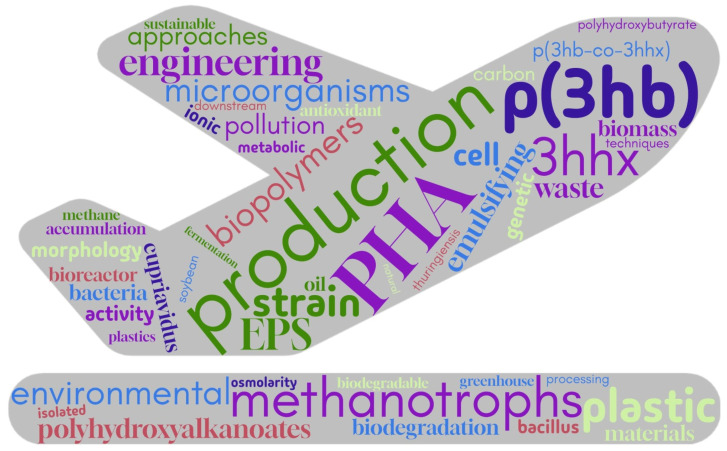
Word cloud of articles based on the theme of the Special Issue. The most frequent words are displayed with a larger font.

## Abbreviations

The following abbreviations are used in this manuscript:

PHAs	Polyhydroxyalkanoates
PHB	Polyhydroxybutyrate
EPSs	Exopolysaccharides
SDG	Sustainable Development Goal
WCO	Waste Cooking Oil
MMC	Mixed Microbial Culture
